# Impact of time from diagnosis to endoscopic submucosal dissection on curability in superficial esophageal squamous cell carcinoma

**DOI:** 10.1002/deo2.70035

**Published:** 2024-11-12

**Authors:** Daiki Sato, Maasa Sasabe, Tomohiro Mitsui, Yasuaki Furue, Takako Yoshii, Hiroki Hara, DaiJi Oka, Takashi Fukuda, Yusuke Yoda

**Affiliations:** ^1^ Department of Endoscopy Saitama Cancer Center Saitama Japan; ^2^ Department of Gastroenterology Saitama Cancer Center Saitama Japan; ^3^ Department of Gastroenterological Surgery Saitama Cancer Center Saitama Japan

**Keywords:** curability, delayed treatment, endoscopic submucosal dissection, retrospective study, superficial esophageal squamous cell carcinoma

## Abstract

**Objective:**

To investigate the time delay effect from initial diagnosis to endoscopic submucosal dissection on superficial esophageal squamous cell carcinoma curability, considering the preoperative invasion depth.

**Methods:**

This study included superficial esophageal squamous cell carcinoma diagnosed as T1a‐epithelial/lamina propria mucosa cancer (cEP/LPM; cancer invading up to the lamina propria mucosa) or cT1a‐muscularis mucosa (MM)/T1b‐submucosal cancer (cMM/SM1; cancer invading up to 200 µm into the submucosa) and treated using endoscopic submucosal dissection from January 2017 to December 2021. We compared curability in lesions treated within three months (early treatment group) versus those treated ≥7 months post‐diagnosis (delayed treatment group). Curative resection criteria included lesions confined within the muscularis mucosae, with negative vertical margins, and with absence of lymphovascular invasion. Non‐curative resection included all other cases.

**Results:**

Among the 231 and 75 lesions in the early and delayed treatment groups, respectively, no significant difference was observed in non‐curative resections for all lesions and cEP/LPM lesions (early: 194, delayed: 70). Conversely, the proportions were significantly higher in the delayed treatment group than in the early treatment group for cMM/SM1 lesions (early: 37, delayed: 5; *p* = 0.018).

**Conclusions:**

This study suggests that delayed endoscopic submucosal dissection does not significantly affect cEP/LPM lesions curability but is associated with reduced cMM/SM1 lesions curability. Prompt treatment is important for cMM/SM1, whereas delayed treatment may be acceptable for cEP/LPM.

## INTRODUCTION

Endoscopic submucosal dissection (ESD) is widely utilized to treat superficial esophageal squamous cell carcinoma (SESCC) with a low lymph node metastasis (LNM) risk.^1^, ^2^ ESD allows en‐bloc resection, facilitating accurate pathological evaluation, regardless of lesion size. A comprehensive pathological assessment post‐ESD is vital for determining SESCC curability. Non‐curative resection prompts additional treatments owing to the LNM risk.^3^, ^4^, ^5^

Preoperative clinical assessment of cancer invasion depth strongly correlates with LNM risk and is critical to ESD indications. Clinically, ESD is typically indicated for T1a‐epithelial/lamina propria (cEP/LPM) cancers owing to its high accuracy (approximately 90%) for cEP/LPM invasion depth diagnosis and the exceedingly low LNM risk in pathological (p) EP/LPM cancers.^2^, ^6^ Conversely, cT1a‐muscularis mucosa (MM)/T1b‐submucosa (≤200 µm, SM1) cancers have increased LNM risk, and the preoperative cancer invasion depth diagnostic accuracy for cMM/SM1 is insufficient.^1^ Therefore, the potential non‐curative resection risk and the need for additional treatments should be considered when treating cMM/SM1 lesions via ESD.

Immediate treatment is desirable after diagnosing SESCC with ESD indications, albeit not always possible. Therefore, investigating whether the time from the initial diagnosis to ESD treatment affects SESCC curability is important.

No previous studies have explored the relationship between the time from the initial diagnosis to ESD and SESCC curability. Therefore, this study aimed to elucidate this relationship.

## METHODS

Detailed descriptions of endoscopic invasion depth initial diagnosis, ESD procedure, and pathologic evaluation are presented in Appendix S1.

### Study design and patients

This retrospective cohort study was conducted at a tertiary cancer center. We analyzed the medical records of consecutive patients with SESCC diagnosed as cEP/LPM or cMM/SM1 (N0M0) and indicated ESD between June 2017 and December 2021.

The exclusion criteria included lesions in which the invasion depth was difficult to assess owing to their location on ESD scars or residual disease after chemoradiotherapy for ESCC.

We confirmed whether these lesions had progressed to surgery or CRT during the follow‐up; lesions that underwent ESD were divided into two groups for analysis based on the time from the initial diagnosis to ESD: early and delayed treatment groups.

The duration for the early and delayed treatment groups was defined based on our institution's follow‐up intervals by endoscopy. Typically, lesions indicated for ESD were treated within three months. However, when immediate treatment was not feasible, endoscopy was often performed approximately every six months to assess the lesion status based on common practice determined at the attending physician's discretion. Therefore, the early treatment group was defined as lesions treated within three months, whereas the delayed group was defined as lesions treated ≥7 months post‐diagnosis, where ESD was performed after at least one six‐month follow‐up and subsequent hospital admission.

Metachronous cancer was defined as SESCC that occurred at a site different from the first location and at a time different from that of the first endoscopic resection for SESCC. Other concurrent organ cancers were defined as cancers found in organs other than the esophagus at the time the index SESCC lesion was diagnosed.

### Initial ESCC diagnosis and endoscopic invasion depth

The initial ESCC diagnosis date was defined as either the date when ESCC was diagnosed endoscopically at our institution or the date when the biopsy was performed in cases of diagnosis via endoscopic biopsy at our institution. Endoscopic ESCC diagnosis was defined as lesions with type B vessels endoscopic findings, based on the magnifying endoscopic classification of the Japan Esophageal Society.^7^ Lesions where the vasculature could not be observed were diagnosed through biopsy.

To evaluate the clinical invasion depth of the lesion, we adopted a commonly used approach involving endoscopic diagnosis based on three categories: cEP/LPM, cMM/SM1, and cSM2. In cases where determining the invasion depth was challenging using both non‐magnified and magnified endoscopy, endoscopic ultrasound was employed to assist in the evaluation.

### Curability assessment

Curability was assessed based on the histological findings following ESD, and the curability criteria were defined according to Japanese EMR/ESD guidelines and Japanese practice guidelines for esophageal cancer.^1^, ^8^ Curative resection was defined as lesions histologically confined within the muscularis mucosae, with negative vertical margins, and with no lymphovascular invasion, whereas non‐curative resection was defined as all other cases. Additionally, high‐risk non‐curative resection was defined as lesions with pT1b‐SM2 or lymphovascular invasion. Lesions invading the muscularis mucosae without lymphovascular invasion were included in the curative resection because the lymph node metastasis risk was considered low (approximately 5%), and no additional treatment recommendation was determined.^1^

### Statistical analyses

The primary outcome was the curability of the two groups based on their preoperative invasion depths (cEP/LPM and MM/SM1) determined at the initial diagnosis. The time from diagnosis to treatment was defined as the time from the SESCC diagnosis date to ESD at our institution.

Categorical data were expressed as frequencies (%) and compared using Fisher's exact test. Continuous data were expressed as medians with ranges and compared using the Mann–Whitney U test.

All statistical analyses were performed using EZR (Saitama Medical Center, Jichi Medical University), a graphical user interface for R (R Foundation for Statistical Computing). All *p*‐values were two‐sided, and statistical significance was set at *p* < 0.05.

## RESULTS

### Patient and lesion characteristics at initial diagnosis

**FIGURE 1:**
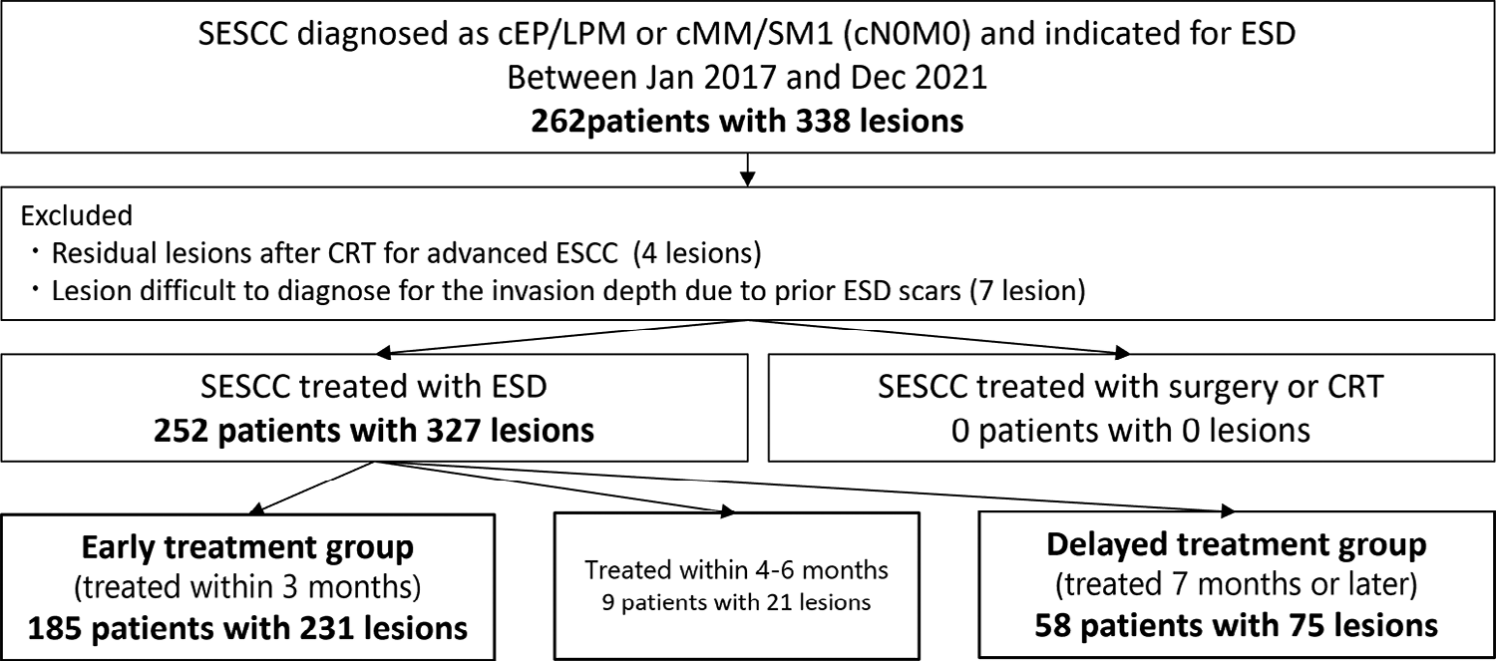
**Patient and lesion inclusion flow diagram**. cEP/LPM, clinically T1a‐epithelial/lamina propria; cMM/SM1, cT1a‐muscularis mucosa/T1b‐submucosa 1 (cancer invading ≤ 200 µm into the submucosa); ESD, endoscopic submucosal dissection; SESCC, superficial esophageal squamous cell carcinoma.

Figure 1 demonstrates a flow diagram of the included patients and lesions. In this study, there were no lesions that progressed to surgery or chemoradiotherapy during the treatment course. Ultimately, 252 patients with 327 lesions who underwent ESD were included in the analysis. Overall, 185 and 58 patients with 231 and 75 lesions were included in the early and delayed treatment groups, respectively. Additionally, 9 patients with 21 lesions underwent ESD 4–6 months after the initial diagnosis.

Table 1 presents comparisons of patient and lesion characteristics at the initial diagnosis between the groups. The proportion of lesions with metachronous cancer was significantly higher in the delayed treatment group than in the early treatment group (61.3% vs. 35.1%; *p* < 0.001). The median endoscopic tumor size was smaller in the delayed treatment group than in the early treatment group (10 mm vs. 15 mm; *p* < 0.001), whereas the macroscopic type and circumference were not significantly different between the groups.

**TABLE 1:** Patient and lesion characteristics at the initial diagnosis.^†^

	All lesions			cEP/LPM at the initial diagnosis			cMM/SM1 at the initial diagnosis		
Patients^‡^	Early treatment group (*n* = 185)	Delayed treatment group (*n* = 58)	*p*‐value	Early treatment group *(n* = 156)	Delayed treatment group (*n* = 53)	*p*‐value	Early treatment group (*n* = 35)	Delayed treatment group (*n* = 5)	*p*‐value
Sex: *n* (%)			**0.0291**			0.0932			0.5628
Male	154 (83.2)	55 (94.8)		127 (84.7)	50 (94.3)		27 (77.1)	5 (100)	
Female	31 (16.8)	3 (5.2)		23 (15.3)	3 (5.7)		8 (22.9)	0 (0)	
Median age, years (range)	71 (44–87)	70 (49–83)	0.6	71 (45–87)	70 (49–83)	0.49	71 (44–83)	73 (60–78)	0.87

Lesions	Early treatment group (*n* = 231)	Delayed treatment group (*n* = 75)	*p*‐value	Early treatment group (*n* = 194)	Delayed treatment group (*n* = 70)	*p*‐value	Early treatment group (*n* = 37)	Delayed treatment group (*n* = 5)	*p*‐value
Primary or metachronous ESCC, *n* (%)			**<0.001**			**<0.001**			1
Primary	150 (64.9)	29 (38.7)		122 (62.9)	25 (35.7)		28 (75.7)	4 (80)	
Metachronous	81 (35.1)	46 (61.3)		81 (37.1)	45 (64.3)		9 (24.3)	1 (20)	
Endoscopic tumor size, median (range), mm	15 (3–70)	10 (3–50)	**<0.001**	15 (3–70)	10 (3–50)	**<0.001**	15 (3–70)	30 (8–45)	0.0632
Tumor location, *n* (%)			0.994			0.987			0.838
Upper (Ce, Ut)	48 (20.8)	14 (18.6)		38 (19.6)	13 (18.6)		10 (27.0)	1 (20)	
Middle (Mt)	118 (51.1)	40 (53.3)		100 (51.5)	38 (54.3)		18 (48.6)	2 (40)	
Lower (Lt, Ae)	65 (28.1)	21 (28.0)		56 (28.9)	19 (27.1)		9 (24.3)	2 (40)	
Macroscopic type, *n* (%)			0.678			1			1
0‐IIa	4 (1.7)	0 (0)		1 (0.5)	0 (0)		3 (8.1)	0 (0)	
0‐IIc	219 (94.8)	75 (100)		192 (99)	70 (100)		27 (73.0)	5 (100)	
Mixed	8 (3.5)	0 (0)		1 (0.5)	0 (0)		7 (18.9)	0 (0)	
Circumference, *n* (%)			1			1			1
Less than ¾	222 (96.1)	73 (97.3)		191 (98.5)	69 (98.6)		31 (83.8)	4 (80)	
¾‐ near total	9 (3.9)	2 (2.7)		3 (1.5)	1 (1.4)		6 (16.2)	1 (20)	

Abbreviation: ESCC, esophageal squamous cell carcinoma.

^†^ Values are median (range) or *n* (%).

^‡^ “Patients” includes duplicates, as some patients had metachronous cancers.

cEP/LPM lesions had a significantly higher proportion of lesions with metachronous cancer (37.1% vs. 64.3%; *p* < 0.001) and a significantly smaller median endoscopic tumor size in the delayed treatment group than in the early treatment group (10 vs. 15 mm; *p* < 0.001). No significant differences were observed in cMM/SM1 lesions.

### Endoscopy timing and frequency from initial diagnosis to ESD, treatment delay reasons, and concurrent other organ cancers

The median times from the initial diagnosis to ESD were one and 11 months in the early and delayed treatment groups, respectively, for all lesions, with 11.5 and nine months for cEP/LPM and cMM/SM1 lesions, respectively, in the delayed treatment group (Table 2). In the delayed treatment group, the median endoscopy interval from the initial diagnosis to ESD was 6.5 months, with an 18‐month maximum interval. The lesion with the longest 18‐month interval was initially diagnosed as cEP/LPM. The patient had concurrent head and neck cancer requiring surgery, and endoscopic evaluation was performed after treatment stabilization. The lesion with the longest time to ESD (46 months) was initially diagnosed as EP/LPM. The delay was due to the patient's inability to quit drinking, with an endoscopic evaluation performed every 5.5 months.

**TABLE 2:** Timing and frequency of endoscopy from initial diagnosis to endoscopic submucosal dissection.^†^

	All lesions		cEP/LPM at the initial diagnosis		cMM/SM1 at the initial diagnosis	
Lesions	Early treatment group (*n* = 231)	Delayed treatment group (*n* = 75)	Early treatment group (*n* = 194)	Delayed treatment group (*n* = 70)	Early treatment group (*n* = 37)	Delayed treatment group (*n* = 5)
Time from initial diagnosis to ESD, median (range), month	1 (0–3)	11 (7–46)	1 (0–3)	11.5 (8–46)	1 (0–3)	9 (7–14)
Number of endoscopies performed from initial diagnosis to ESD, median (range)	0 (0–1)	2 (1–8)	0 (0–1)	2 (1–8)	0 (0)	1 (1–2)
Endoscopy interval within the period from initial diagnosis to ESD, median (range), month		6.5 (3–18)		6.5 (3–18)		9 (3.5–14)

Abbreviation: ESD, endoscopic submucosal dissection.

^†^ Values are median (range) or *n* (%).

The most common reason for delay in all patients was treatment for concurrent other organ cancers (37.9%), followed by inability to quit drinking (34.9%) owing to concerns regarding poor sedation and difficulties during ESD from inflammation with alcohol consumption (Table 4). For sedation, we primarily used a combination of benzodiazepines and opioids, tailored to the patient's condition. Among patients with cEP/LPM, the inability to quit drinking was the most frequent (35.8%) reason. For patients with cMM/SM1, 80% prioritized treatment for concurrent other organ cancers. Regarding concurrent other organ cancers, head and neck cancer was the most common among all patients (Table 3).

**TABLE 3:** Details on concurrent other organ cancers.^†^

	All lesions		cEP/LPM at the initial diagnosis		cMM/SM1 at the initial diagnosis	
Patients with concurrent other organ cancer	Early treatment group (*n* = 12)	Delayed treatment group (*n* = 22)	Early treatment group (*n* = 11)	Delayed treatment group (*n* = 18)	Early treatment group (*n* = 1)	Delayed treatment group (*n* = 4)
Treatment for concurrent other organ cancers, *n* (%)	12 (100)	22 (100)	11 (100)	18 (100)	1 (100)	4 (100)
Type of concurrent other organ cancers, *n* (%)						
Head and neck cancer	10 (83.3)	19 (86.4)	9 (81.8)	16 (88.9)	1 (100)	3 (75)
Gastric cancer	2 (16.7)	1 (4.5)	2 (18.2)	1 (5.6)	0 (0)	0 (0)
Colon cancer	0 (0)	2 (9.1)	0 (0)	1 (5.6)	0 (0)	1 (25)
Stage, *n* (%)						
I	4 (33.3)	4 (18.2)	4 (36.4)	4 (22.0)	0 (0)	0 (0)
II	4 (33.3)	4 (18.2)	4 (36.4)	4 (22.0)	0 (0)	0 (0)
III	3 (25)	7 (27.2)	2 (18.2)	5 (27.8)	1 (100)	2 (50)
IV	1 (8.4)	8 (36.4)	1 (9.1)	5 (27.8)	0 (0)	2 (50)
Treatment, *n* (%)						
Surgery alone	9 (75)	8 (36.4)	8 (72.8)	7 (38.9)	1 (100)	1 (25)
Surgery+chemothrapy or CRT or RT	0 (0)	5 (22.7)	0 (0)	4 (22.0)	0 (0)	1 (25)
CRT	0 (0)	8 (36.4)	0 (0)	7 (38.9)	0 (0)	1 (25)
RT	3 (25)	1 (4.5)	3 (27.2)	0 (0)	0 (0)	1 (25)
R0 resection or complete response, *n* (%)						
Yes	12 (100)	22 (100)	11 (100)	18 (100)	1 (100)	4 (100)
No	0 (0)	0 (0)	0 (0)	0 (0)	0 (0)	0 (0)

Abbreviations: CRT, chemoradiotherapy; Surgery, ???; RT, radiotherapy.

^†^ Values are median (range) or *n* (%).

**TABLE 4:** Reasons for treatment delay.

	Delayed treatment group		
Patients	All (*n* = 58)	cEP/LPM at the initial diagnosis (*n* = 53)	cMM/SM1 at the initial diagnosis (*n* = 5)
Treatment for concurrent other organ cancers, *n* (%)	22 (37.9)	18 (34)	4 (80)
Inability to quit drinking, *n* (%)	20 (34.5)	19 (35.8)	1 (20)
Patient preference, *n* (%)	1 (1.7)	1 (1.4)	0 (0)
Treatment for benign diseases, *n* (%)	1 (1.7)	1 (1.4)	0 (0)
Unknown, *n* (%)	14 (24.2)	14 (26.4)	0 (0)

### Pathological results following ESD

Table 5 presents the pathological results of ESD. No significant differences were observed in invasion depth, en‐bloc resection proportion, negative margin proportion, and lymphovascular invasion proportion between the groups. The difference between endoscopic tumor size at the initial diagnosis and pathologic tumor size was significantly larger in the delayed treatment group than in the early treatment group (4 mm vs. 1 mm; *p* = 0.0172).

**TABLE 5:** Pathological results.^†^

	All lesions			cEP/LPM at the initial diagnosis			cMM/SM1 at the initial diagnosis		
Lesions	Early treatment group (*n* = 231)	Delayed treatment group (*n* = 75)	*p*‐value	Early treatment group (*n* = 194)	Delayed treatment group (*n* = 70)	*p*‐value	Early treatment group (*n* = 37)	Delayed treatment group (*n* = 5)	*p*‐value
Tumor size, median (range), mm	18 (2–70)	17 (4–57)	0.115	18.5 (4–70)	16 (4–45)	**0.015**	15 (2–68)	40 (14–57)	**0.0291**
Difference between endoscopic tumor size at initial diagnosis and pathologic tumor size, median (range), mm	1 (‐19–30)	4 (‐15–32)	**0.0172**	1 (‐19–30)	4 (‐15–32)	**0.0247**	1 (‐15–27)	6 (‐5–17)	0.38
Invasion depth, *n* (%)			0.887			0.761			0.166
pEP	65 (28.1)	24 (32.0)		64 (33)	24 (34.3)		1 (2.7)	0 (0)	
pLPM	118 (51.1)	37 (49.3)		110 (56.7)	37 (52.9)		8 (21.6)	0 (0)	
pMM	32 (13.9)	8 (10.7)		13 (6.7)	7 (10)		19 (51.4)	1 (20)	
pSM1 (submucosa ≤200 µm)	7 (3.0)	2 (2.7)		3 (1.5)	0 (0)		4 (10.8)	2 (40)	
pSM2 (submucosal >200 µm)	9 (3.9)	4 (5.3)		4 (2.1)	2 (2.9)		5 (13.5)	2 (40)	
En‐bloc resection, *n* (%)	237 (100)	75 (100)	1	194 (100)	70 (100)	1	37 (100)	5 (100)	1
Horizontal margin, *n* (%)			0.0765			0.097			0.557
Negative	197 (85.3)	57 (76)		165 (85.1)	53 (75.7)		32 (86.5)	4 (80)	
Positive	30 (13.0)	12 (16)		26 (13.4)	11 (15.7)		4 (10.8)	1 (20)	
Unknown	4 (1.7)	6 (8)		3 (1.5)	6 (8.6)		1 (2.7)	0 (0)	
Vertical margin, *n* (%)			0.571			1			0.226
Negative	229 (99.1)	74 (98.7)		193 (99.5)	70 (98.7)		36 (97.3)	4 (80)	
Positive	1 (0.4)	0 (0)		0 (0)	0 (0)		1 (2.7)	0 (0)	
Unknown	1 (0.4)	1 (1.3)		1 (0.5)	0 (0)		0 (0)	1 (20)	
Lymphatic invasion, *n* (%)			0.796			1			**0.0259**
Negative	215 (93.1)	69 (92)		187 (96.4)	68 (97.1)		28 (75.7)	1 (20)	
Positive	16 (6.9)	6 (8)		7 (3.6)	2 (2.9)		9 (24.3)	4 (80)	
Vascular invasion, *n* (%)			0.267			1			**0.0259**
Negative	225 (97.4)	71 (94.3)		192 (99)	69 (99.6)		33 (89.2)	2 (40)	
Positive	6 (2.6)	4 (5.3)		2 (1)	1 (1.4)		4 (10.8)	3 (60)	

^†^ Values are median (range) or *n* (%).

Regarding cEP/LPM lesions, the median tumor size was significantly larger in the early treatment group than in the delayed treatment group (18.5 mm vs. 16 mm; *p* = 0.015), whereas the difference between endoscopic tumor size at the initial diagnosis and pathologic tumor size was significantly larger in the delayed treatment group than in the early treatment group (4 mm vs. 1 mm; *p* = 0.0247). Regarding cMM/SM1 lesions, the median tumor size was significantly larger in the delayed treatment group than in the early treatment group (40 mm vs. 15 mm; *p* = 0.0291). No significant differences in pathological invasion depth were observed between the two groups; however, lymphatic and vascular invasion proportions were significantly higher in the delayed treatment group than in the early treatment group (lymphatic: 24.3% vs. 80%, *p* = 0.0259; vascular: 10.8% vs. 60%, *p* = 0.0259).

### Curability

Table 6 demonstrates a comparison of curability. No significant difference was observed in the proportion of non‐curative and high‐risk non‐curative resections between the groups for all lesions (early vs. delayed: 11.7% vs. 12%; 9.1% vs. 12%) and cEP/LPM lesions (early vs. delayed: 6.2% vs. 5.7%; 5.1% vs. 5.7%).

**TABLE 6:** Curability.

	All lesions			cEP/LPM at the initial diagnosis			cMM/SM1 at the initial diagnosis		
Lesions	Early treatment group (*n* = 231)	Delayed treatment group (*n* = 75)	*p*‐value	Early treatment group (*n* = 194)	Delayed treatment group (*n* = 70)	*p*‐value	Early treatment group (*n* = 37)	Delayed treatment group (*n* = 5)	*p*‐value
Non‐curative resection, *n* (%)	27 (11.7)	9 (12)	1	27 (6.2)	4 (5.7)	1	15 (40.5)	5 (100)	**0.018**
High‐risk non‐curative resection, *n* (%)	21 (9.1)	9 (12)	0.509	9 (5.1)	4 (5.7)	0.7592	12 (31.6)	5 (100)	**0.0107**

However, for cMM/SM1 lesions at initial diagnosis, the proportions of non‐curative and high‐risk non‐curative resections were significantly higher in the delayed treatment group than in the early treatment group (40.5% vs. 100%, *p* = 0.018 and 31.6% vs. 100%, *p* = 0.01, respectively). Although no cases required conversion to surgery or chemoradiotherapy despite treatment delays, four lesions in the delayed treatment group progressed to cSM2, having been initially diagnosed as cEP/LPM (*n* = 2) or cMM/SM1 (*n* = 2). While these lesions were no longer indicated for ESD, it was performed in all four cases due to patient conditions, resulting in non‐curative resections. One of the four lesions involved a 6 mm cEP/LPM lesion in the cervical esophagus, within the irradiation field for CRT for concurrent hypopharyngeal cancer. After CRT, the lesion became indistinct but was diagnosed as a 6 mm IIa+IIc lesion 21 months later. This was the only lesion within the irradiation field in this study.

**FIGURE 2:**
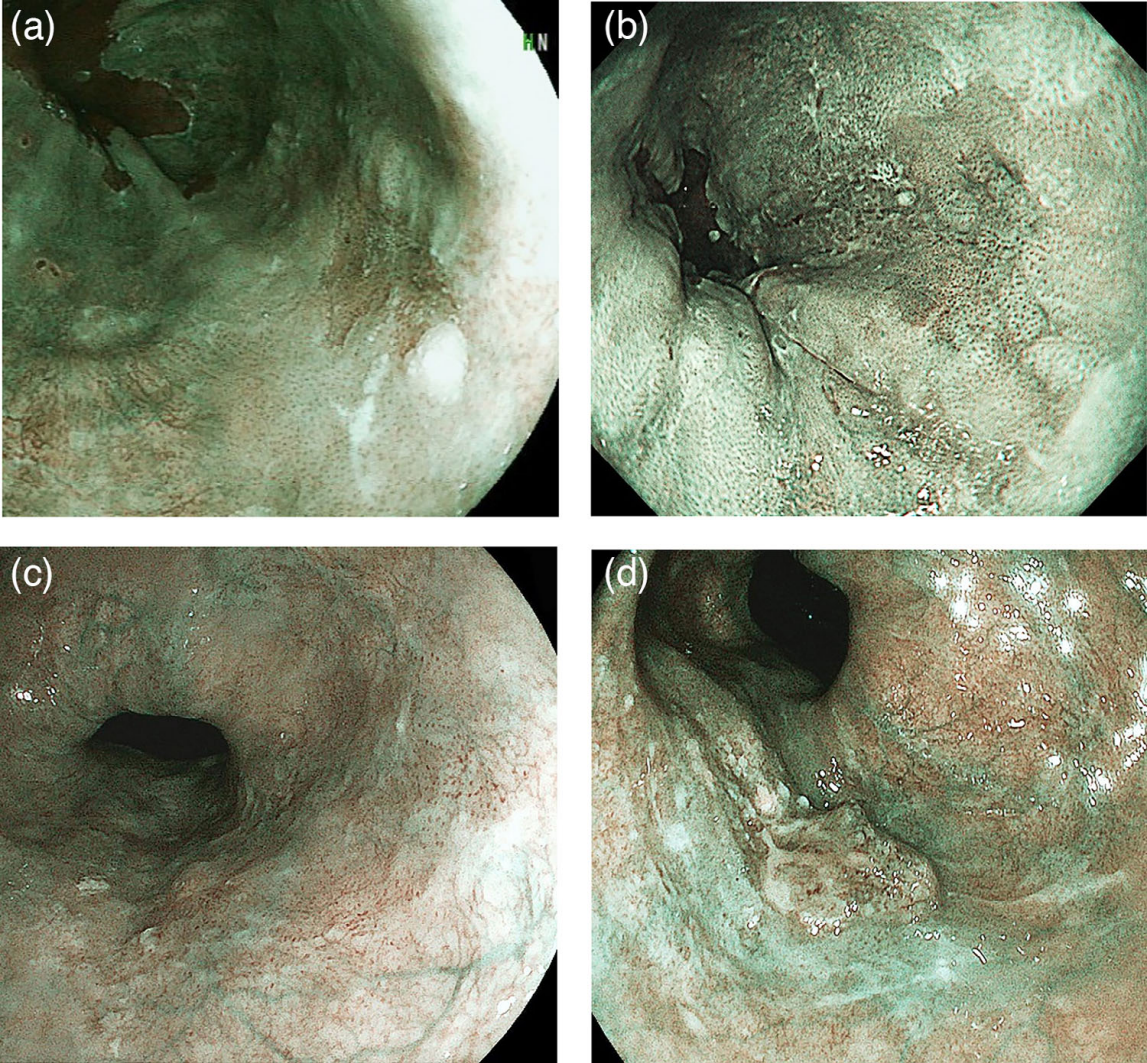
**Representative cases of the course of cEP/LPM and cMM/SM1 lesions at the initial diagnosis in the delayed treatment group**. (a, b) and (c, d) show the cEP/LPM and cMM/SM1 cases in the delayed treatment group, respectively. (a) An initial endoscopic examination with non‐magnifying BLI identifies a flat lesion as a brownish area on the right wall of the lower esophagus. Based on the findings from both non‐magnifying and magnifying observations, the lesion was diagnosed as a cEP/LPM. (b) After 46 months. The lesion increased in size with more distinct boundaries. Post‐ESD pathological findings confirmed pLPM without lymphovascular invasion, and curative resection was performed. (c) Initial endoscopic examination with non‐magnifying BLI reveals a flat lesion with a slight central protrusion and a shallow depression in the posterior wall of the lower esophagus. Based on the findings of both non‐magnifying and magnifying observations, the lesion was diagnosed as cMM/SM1. (d) After 8 months. The center of the lesion clearly shows protrusions. Post‐ESD pathological findings confirmed pSM2 with venous invasion, resulting in high‐risk non‐curative resection. cEP/LPM, clinically T1a‐epithelial/lamina propria; cMM/SM1, cT1a‐muscularis mucosa /T1b‐submucosa 1 (cancer invading ≤ 200 µm into the submucosa); ESD, endoscopic submucosal dissection; pSM2, pathologically submucosa 2 (cancer invading > 200 µm into the submucosa).

Figure 2 demonstrates representative cases of the course of cEP/LPM and cMM/SM1 lesions at initial diagnosis in the delayed treatment group.

Table 7 presents the relationship between tumor size and curability. Regarding the median endoscopic tumor size at the initial diagnosis, the median sizes in cEP/LPM for non‐curative and high‐risk non‐curative resections were significantly larger than those with curative resection (27.5 mm/30 mm vs. 15 mm, *p* = 0.0498/0.0293). The median pathological tumor size in cEP/LPM lesions was significantly larger in the non‐curative resection group compared to the curative resection group (24.5 mm vs. 17 mm; *p* = 0.0448).

**TABLE 7:** Relationship between tumor size and curability.

	Curative resection	Non‐curative resection/ High‐risk non‐curative resection	*p*‐value
Endoscopic tumor size at the initial diagnosis median (range), mm			
All lesions	15 (3–70)	20 (3–70)/ 20 (3–70)	0.0741/0.0609
cEP/LPM	15 (3–70)	27.5 (6–70)/ 30 (6–70)	**0.0498/0.0293**
cMM/SM1	15 (3–40)	15 (6–70)/ 20 (6–70)	0.0694/0.267
Pathological tumor size, median (range), mm			
All lesions	17 (2–70)	20.5 (3–68)/22 (3–68)	0.212/0.0934
cEP/LPM	17 (4–70)	24.5 (6–58)/23 (6–58)	**0.0448**/0.0702
cMM/SM1	16.5 (2–37)	16.0 (3–66)/20 (3–68)	0.0694/0.243
Difference between endoscopic tumor size at initial diagnosis and pathologic tumor size, median (range), mm			
All lesions	2 (−19–32)	1 (−15–32)/1.5 (−15–32)	0.0741/0.0609
cEP/LPM	2 (−19–32)	0 (−12–10)/0 (−12–10)	0.0741/0.163
cMM/SM1	0.5 (−5–27)	2 (−15–17)/2 (−15–17)	0.294/0.241

## DISCUSSION

ESD delays did not significantly affect curability for lesions initially diagnosed as cEP/LPM, even with delays exceeding seven months from diagnosis to ESD. Contrastingly, such delays were associated with reduced curability for cMM/SM1 lesions, suggesting that, while delays in ESD might be acceptable for cEP/LPM lesions, prompt intervention is crucial for cMM/SM1 lesions. To the best of our knowledge, no studies have investigated the effect of ESD delay on SESCC curability.

Here, the impact of treatment delay on curability varied depending on the depth of endoscopic invasion in SESCC. Generally, endoscopic depth diagnosis for SESCC is categorized into three groups: cEP/LPM, cMM/SM1, and cSM2, with cEP/LPM and cMM/SM1 considered suitable for endoscopic resection. Particularly, cEP/LPM lesions are considered good candidates for ESD owing to their low metastatic risk, and the invasion depth can be accurately diagnosed via ESD, with an accuracy of approximately 90%. Contrastingly, ESD has inadequate depth diagnostic accuracy for cMM/SM1 lesions (approximately 55%).^1^ Herein, the accuracies of invasion depth diagnosis for cEP/LPM and cMM/SM1 lesions at the initial diagnosis in the early treatment group were 90% and 62.2%, respectively, similar to previous reports.^1^ This consistency in diagnostic accuracy supports the validity of our findings, suggesting the reliability of our results regarding the treatment timing effect on curability.

The curative resection rate was approximately 94% for lesions diagnosed as cEP/LPM at initial diagnosis in both groups, without significant differences. Here, factors other than the duration until ESD may have influenced curability, including the initial endoscopic and pathological tumor size, which are both smaller in the delayed treatment group (median endoscopic size early vs. delayed: 15 mm vs. 10 mm, and pathological size early vs. delayed: 18.5 mm vs. 16 mm). In other words, although the period from diagnosis to treatment was prolonged, the lesions, being smaller in size, may have contributed to the lack of significant differences in curability. Regarding the relationship between lesion size and curability, a single‐center retrospective study reported that larger lesion sizes, especially those larger than 50 mm, decreased the diagnostic accuracy of preoperative invasion depth and increased non‐curative resections, even for lesions diagnosed as cEP/LPM.^6^ The median initial endoscopic tumor size and pathological sizes in cEP/LPM for non‐curative resections were considerably larger than those with curative resections (non‐curative vs. curative; 27.5 mm vs. 15 mm for endoscopic size, and 24.5 mm vs. 17 mm for pathological size), as shown in Table 7 and Table S1. Additionally, nearly half of these lesions were close to 50 mm pathologically, consistent with previous reports. Therefore, the treatment strategy should consider the possibility of non‐curative resection for larger lesion sizes, especially those larger than 50 mm, even if a lesion is diagnosed as cEP/LPM.

In contrast to lesions initially diagnosed as cEP/LPM, those diagnosed as cMM/SM1 exhibited markedly higher proportions of non‐curative and high‐risk non‐curative resections in the delayed treatment group than in the early treatment group. This contrast can be attributed to the fact that approximately 70% of cMM/SM1 lesions are pathologically diagnosed as extending beyond the muscularis mucosae after ESD,^1^ indicating a higher invasive potential than that of cEP/LPM lesions. Additionally, the esophagus anatomy, with abundant lymphatic and blood vessels in the submucosal layer,^9^ suggests an increased risk of lymphovascular invasion. A systematic review of seven major cancer types, while not focused on early cancers, indicated that a 4‐week treatment delay was associated with increased mortality,^10^ in line with our findings on delay in cMM/SM1 lesions. Notably, no significant difference was observed in invasion depth in the pathological results for cMM/SM1 between the early and delayed treatment groups. However, the proportion of lymphovascular invasion was remarkably higher in the delayed treatment group than in the early treatment group. This suggests that while major changes in invasion depth may be rare within a maximum delay of 14 months from diagnosis to ESD for cMM/SM1 lesions, their anatomical characteristics indicate that a delay of several months may result in subtle changes. The results for cMM/SM1 lesions offer valuable insights, though the limited sample size in the delayed treatment group warrants a cautious interpretation. Nevertheless, our findings emphasize the importance of timely treatment for cMM/SM1 lesions. Particularly when delays occur due to treatments of concurrent other organ cancers, close collaboration with those departments is crucial to minimize delays.

Approximately 30% of the lesions in the delayed treatment group underwent surgery or chemoradiotherapy for concurrent other organ cancers between the initial diagnosis and ESD, including an initial cEP/LPM lesion within the irradiation field for CRT for concurrent hypopharyngeal cancer, which became indistinct after CRT but was diagnosed as a cSM2 lesion 21 months later. This suggests that treatment for concurrent other organ cancers may cause lesions to follow an irregular course, differing from natural progression. Therefore, the results in our study may partly reflect the impact of these treatments in addition to time progression.

Our study had some limitations. First, this was a retrospective study at a single institution. Second, cEP/LPM lesions had a higher proportion of metachronous cancers and more surveillance endoscopy from diagnosis to treatment than cMM/SM1 lesions in the delayed treatment group; therefore, the increased proportion of curative resections in cEP/LPM may be partly attributed to detection bias. Third, the small sample size for cMM/SM1 lesions in the delayed group may have affected the statistical power and precision of the results owing to interobserver variability and sampling error. However, in clinical practice, analyzing multiple delayed cMM/SM1 cases is challenging. Therefore, we believe that our findings have considerable value.

In conclusion, our findings suggest that delayed ESD did not significantly affect cEP/LPM SESCC curability, whereas such delays were associated with reduced cMM/SM1 lesions curability. ESD for SESCC can be delayed owing to social factors and the management of concurrent other organ cancers. Prompt treatment is crucial for cMM/SM1 lesions, whereas a delay may be acceptable for cEP/LPM lesions.

## CONFLICT OF INTEREST STATEMENT

None.

## ETHICS STATEMENT

The protocol was approved by the Institutional Review Board of Saitama Cancer Center (1488).

## PATIENT CONSENT STATEMENT

Informed consent for participation in this study was obtained through an opt‐out option available on the website.

## CLINICAL TRIAL REGISTRATION

N/A

## Supporting information


**APPENDIX S1**: Initial diagnosis of endoscopic invasion depth, endoscopic submucosal dissection (ESD) procedure, and pathologic evaluations.

**TABLE S1**: Summary of lesions with non‐curative resection in cEP/LPM at the initial diagnosis.^†^
^†^ESD, endoscopic submucosal dissection; HM, horizontal margin; VM, vertical margin.
