# Supplementary material for: Impact of time from diagnosis to endoscopic submucosal dissection on curability in superficial esophageal squamous cell carcinoma
Source: DEN Open. 2024 Nov 12;5(1):e70035. doi: 10.1002/deo2.70035 (PMC11555296; doi:10.1002/deo2.70035)
Supplement: Supplementary file 1 — APPENDIX S1: Initial diagnosis of endoscopic invasion depth, endoscopic submucosal dissection (ESD) procedure, and pathologic evaluations. [file DEO2-5-e70035-s001.docx]

**Appendix S1 – Initial diagnosis of endoscopic invasion depth, endoscopic submucosal dissection (ESD) procedure, and pathologic evaluation**

Six endoscopists participated in the diagnostic and therapeutic endoscopic procedures, all of whom are board-certified fellows of the Japan Gastroenterological Endoscopy Society.

**I****nitial diagnosis of endoscopic invasion depth**

To evaluate the depth of the clinical invasion of the lesion, we adopted a commonly used approach involving endoscopic diagnosis based on three categories: cEP/LPM, cMM/SM1, and cSM2. Non-magnifying and magnifying endoscopy with narrow-band imaging or blue light imaging were used to diagnose invasion depth. Endoscopic ultrasonography was used when the invasion depth was difficult to diagnose using these methods. The diagnosis of invasion depth with non-magnifying and magnifying endoscopy was based on the following findings: cEP/LPM, flat lesion without protrusion or depression and type B1 vessels; cMM/SM1, flat lesion with irregular surface and protrusion < 1 mm or shallow depression and type B2 vessels; and cSM2, lesion with protrusion > 1 mm or deep depression and type B3 vessels.^7^ Regarding cases that were difficult to diagnose, a comprehensive final diagnosis was made during the pretreatment conference to determine the invasion depth.

**E****SD procedure**

A single-channel upper gastrointestinal endoscope (GIF Q260J; Olympus, Tokyo, Japan, or EG-L600WR7; Fujifilm, Tokyo, Japan) was used with an electrosurgical unit (VIO-300; ERBE, Tubingen, Germany) and an electrosurgical knife (dual knife KD-650L; Olympus, Tokyo, Japan or Clutch cutter; Fujifilm, Tokyo, Japan). Tumor outlines were identified using iodine staining and narrow-band imaging or blue light imaging, and the marking dots were placed circumferentially outside the tumor margins using an electrosurgical knife. Subsequently, 0.4% sodium hyaluronic acid (MucoUp; Boston Scientific Japan KK, Tokyo, Japan) was injected into the submucosal layer to lift the lesions. After completing the mucosal incision outside the marking dots, submucosal dissection was performed.

**Pathologic evaluation**

Resected ESD specimens were fixed in formalin, cut into 2 mm slices, stained with hematoxylin and eosin, and evaluated according to the Japanese classification of esophageal cancer.^7^ Macroscopic appearance, invasion depth, tumor size, lymphovascular invasion, horizontal margins, and vertical margins were assessed. pT1b ESCC was classified into pT1b-SM1 (invasion depth into the submucosal layer ≤ 200 mm) and pT1b-SM2 (invasion depth into the submucosal layer > 200 mm).
