# Supplementary material for: Impact of time from diagnosis to endoscopic submucosal dissection on curability in superficial esophageal squamous cell carcinoma
Source: DEN Open. 2024 Nov 12;5(1):e70035. doi: 10.1002/deo2.70035 (PMC11555296; doi:10.1002/deo2.70035)
Supplement: Supplementary file 2 — TABLE S1: Summary of lesions with non‐curative resection in cEP/LPM at the initial diagnosis.† †ESD, endoscopic submucosal dissection; HM, horizontal margin; VM, vertical margin. [file DEO2-5-e70035-s002.docx]

Supplemental Table 1. Summary of lesions with non-curative resection in cEP/LPM at the initial diagnosis^†^

| Group | Sex | Age  (y) | Endoscopic  tumor size  (mm) | Macroscopic  type | Circumference | The duration from the initial diagnosis to ESD (M) | Pathological results | | | | | | |
| --- | --- | --- | --- | --- | --- | --- | --- | --- | --- | --- | --- | --- | --- |
|  |  |  |  |  |  |  | Macroscopic type | Tumor size (mm) | Invasion depth | pHM | pVM | Ly | V |
| Early | male | 77 | 70 | 0-IIc | > 1/2, ≤ 3/4 | 3 | 0-IIc | 58 | SM2 | 1 | 0 | 1 | 1 |
| Early | male | 79 | 50 | 0-IIc | > 1/2, ≤ 3/4 | 1 | 0-IIc | 50 | SM1 | 1 | 0 | 0 | 0 |
| Early | male | 63 | 50 | 0-IIc | > 1/2, ≤ 3/4 | 2 | 0-IIc | 43 | SM2 | 1 | x | 0 | 0 |
| Early | male | 87 | 50 | 0-IIc | > 3/4, < 1 | 2 | 0-IIc | 47 | SM2 | 1 | 0 | 0 | 0 |
| Early | female | 86 | 50 | 0-IIc | > 1/2, ≤ 3/4 | 2 | 0-IIc | 45 | SM2 | 1 | 0 | 1 | 0 |
| Early | male | 69 | 35 | 0-IIc | > 1/2, ≤ 3/4 | 0 | 0-IIc | 45 | MM | 0 | 0 | 1 | 0 |
| Early | male | 72 | 30 | 0-IIc | ≤ 1/4 | 1 | 0-IIc | 32 | MM | 0 | 0 | 1 | 1 |
| Early | male | 55 | 30 | 0-IIc | ≤ 1/4 | 1 | 0-IIc | 21 | MM | 0 | 0 | 1 | 0 |
| Early | male | 76 | 25 | 0-IIc | ≤ 1/4 | 1 | 0-IIc | 26 | SM1 | 0 | 0 | 0 | 0 |
| Early | male | 62 | 15 | 0-IIc | ≤ 1/4 | 1 | 0-IIc | 13 | MM | 0 | 0 | 1 | 0 |
| Early | female | 72 | 15 | 0-IIc | ≤ 1/4 | 1 | 0-IIc | 23 | MM | 0 | 0 | 1 | 0 |
| Delayed | male | 65 | 20 | 0-IIc | ≤ 1/4 | 8 | 0-IIc | 9 | MM | 0 | 0 | 1 | 0 |
| Delayed | male | 60 | 10 | 0-IIc | ≤ 1/4 | 7 | 0-IIc | 18 | MM | 0 | 0 | 1 | 0 |
| Delayed | male | 77 | 10 | 0-IIc | ≤ 1/4 | 8 | 0-IIc | 18 | SM2 | 1 | 0 | 0 | 1 |
| Delayed | male | 66 | 6 | 0-IIc | ≤ 1/4 | 21 | 0-IIa+IIc | 6 | SM2 | 0 | 0 | 0 | 0 |

^†^ESD, endoscopic submucosal dissection; HM, horizontal margin; VM, vertical margin
